# A case of spontaneous isolated superior mesenteric arterial dissection with coeliac axis stenosis

**DOI:** 10.5830/CVJA-2022-066

**Published:** 2023-03-10

**Authors:** Kun Ye, Yong Wang, Shengyun Wan

**Affiliations:** Department of General Surgery, 2nd Affiliated Hospital of Anhui Medical University, Hefei, Anhui, China

**Keywords:** spontaneous isolated superior mesenteric arterial dissection, coeliac axis stenosis, isolated visceral arterial dissection

## Abstract

Spontaneous isolated superior mesenteric arterial dissection with coeliac axis stenosis is rare but serious. We report a case of a 54-year-old male with coeliac axis stenosis who presented with acute superior mesenteric arterial dissection, which caused thrombosis of the branches. This is the first report of the full course of treatment using endovascular repair and laparoscopic surgery to deal with spontaneous isolated superior mesenteric arterial dissection combined with coeliac axis stenosis. This approach has been shown to be safe and effective for yielding short-term results.

Spontaneous isolated superior mesenteric arterial dissection (SISMAD) that is not associated with aortic dissection represents 8% of all visceral arterial dissections and has an overall prevalence of 0.06% in cadaveric studies, described for the first time by Bauersfeld in 1947.^1^ In addition, there have been an increasing number of reports since the improvements in technology, and computed tomography (CT) imaging has been in widespread use for abdominal pain.^2^,^3^ Computerised tomography angiogram (CTA) is a valuable diagnostic modality for SISMAD.

Many classification schemes have been developed based on CTA, such as the Yun, Sakamoto and Zerbib classifications. In recent years, Luan’s type^4^ and Qiu’s type^5^ have been developed. However, the condition of the distal branches involved were not included in previous classifications.

Clinical manifestations vary widely depending on the location of the intimal tear, the range of the dissection, the degree of the compromise of the true lumen, and the number of collateral arteries.^6^ Better typing methods may be needed in the future.

According to the previous literature, the therapeutic regimen for patients with SISMAD should be based on clinical symptoms, and conservative management is feasible in most cases. It is recommended that endovascular stenting combined with laparoscopic exploration and/or open surgery could be a reasonable option for symptomatic SISMAD in which peritonitis is present.^7^

The reported incidence of coeliac axis stenosis ranges from 12.5 to 24% in Western populations.^8^ Reported causes of coeliac axis stenosis are arteriosclerosis, injury from catheter manipulation, surgical trauma, Takayasu arteritis, and compression of the coeliac axis by the median arcuate ligament (MAL).^9^

Among the general population, stenosis of the coeliac axis is usually asymptomatic. This is because of the different vascular systems that can provide collateral circulation. Severe stenosis of the coeliac axis is commonly associated with enlargement of the arteries of the pancreatic–duodenal arcade, which supply the coeliac axis via retrograde flow from the superior mesenteric artery (SMA).

Only one SISMAD patient with coeliac axis stenosis has been reported in the literature thus far. This patient had an asymptomatic coeliac axis stenosis and was found to have acquired SISMAD accidentally. To date, this work is the first report of a hybrid approach for SISMAD combined with coeliac axis stenosis.

## Case report

The patient was a 54-year-old male with a history of uncontrolled hypertension who was admitted to hospital with sudden epigastric pain lasting for three days. The abdomen was bulging and there was pain in the whole abdomen and rebound pain in the periumbilical abdomen. Borborygmus was weak (about twice a minute) and no sounds could be heard in the blood vessels.

**Fig. 1:**
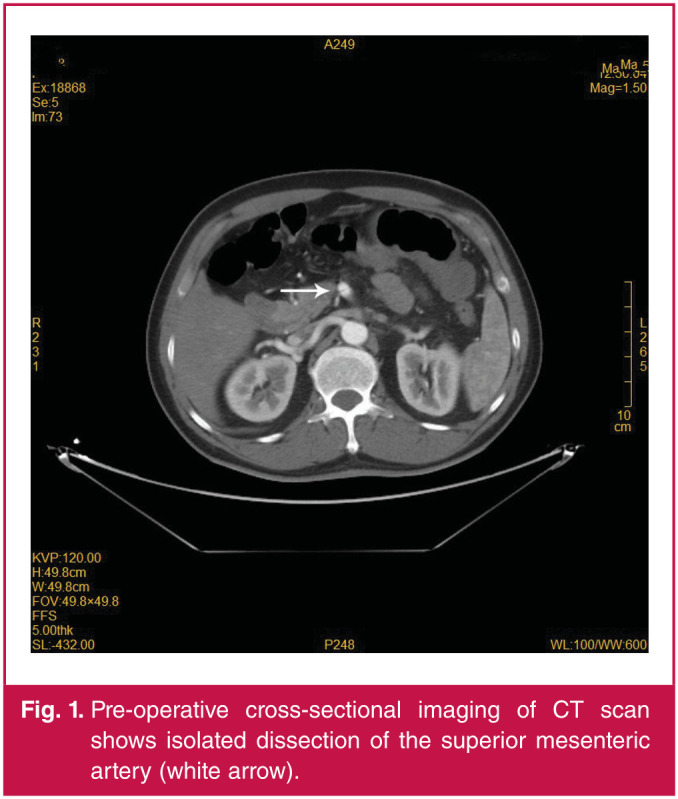
Pre-operative cross-sectional imaging of CT scan shows isolated dissection of the superior mesenteric artery (white arrow).

**Fig. 2:**
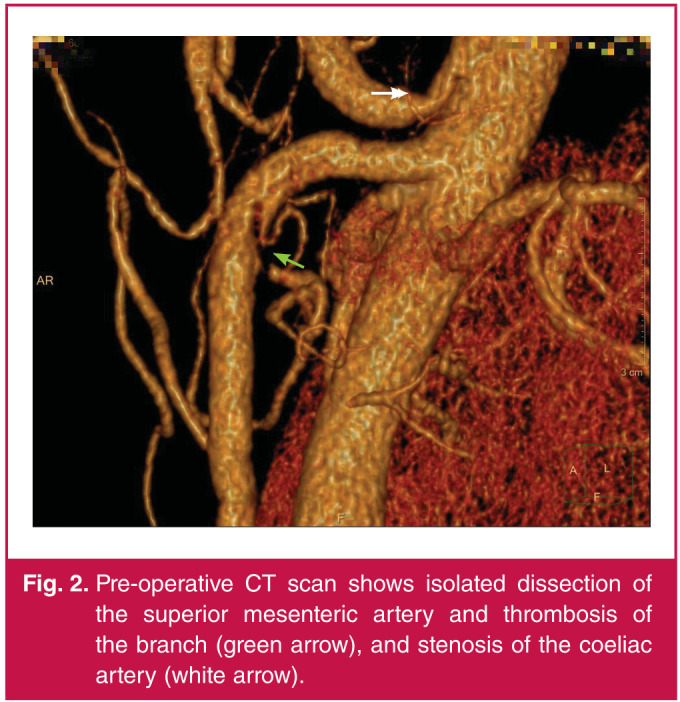
Pre-operative CT scan shows isolated dissection of the superior mesenteric artery and thrombosis of the branch (green arrow), and stenosis of the coeliac artery (white arrow).

CTA of the abdomen showed dissection of the proximal segment of the superior mesenteric artery (Fig. 1), thrombosis in the left branches of the SMA, and stenosis of the coeliac axis (Fig. 2). Hence, the patient was diagnosed with SISMAD (Yun type II) and coeliac axis stenosis.

Informed consent was obtained from the patient, and emergency angiography was performed. Intra-operative angiography showed dissection of the SMA, the intimal tear was 2.5 cm from the ostium, and jejuno-ileal branches were not shown. A stent (8 × 39 mm, Ominlink, Abbott, USA) was successfully implanted to seal off the dissection, revealing the proximal jejunal branches.

Intra-operative angiography also showed severe stenosis of the coeliac axis, and another stent (9 × 29 mm, Ominlink, Abbott, USA) was successfully implanted to expand the stenosis. Soon afterwards, laparoscopy showed no bloody peritoneal effusion, no intestinal necrosis, poor jejuno-ileal blood supply and slow intestinal peristalsis.

Epigastric pain was relieved postoperatively, and the patient was discharged smoothly. However, two months later, the patient was re-admitted to hospital due to severe flatulence after meals. He was characterised by left lower abdominal distension, which was obviously relieved after fasting, accompanied by nausea and vomiting but no abdominal pain or diarrhoea. CTA showed a swollen and dilated jejuno-ileal section due to thrombosis in the left branches of the SMA.

**Fig. 3:**
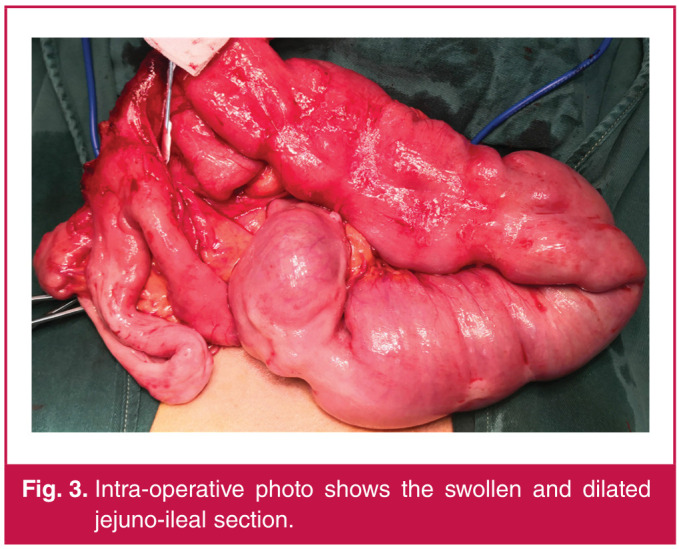
Intra-operative photo shows the swollen and dilated jejuno-ileal section.

After communication with the patient, a second-look laparoscopy was performed. Approximately 60 cm of the jejunoileal region starting 20 cm from the Treitz ligament was swollen. Peristalsis in this region was slow, it had a ruddy appearance, and part of the omentum supplied its blood supply. The swollen and dilated jejuno-ileal section was removed, and side-to-side anastomosis of the normal intestine was performed (Fig. 3). Meanwhile, a jejunal nutrient tube was placed through the nose to the distal end of the anastomosis.

Postoperative pathology indicated ischaemic intestinal changes. After enteral nutrition was given, the patient recovered well and resumed a normal diet one week after the operation.

## Discussion

The treatment strategy of SISMAD needs to be developed according to change in condition of the patient. Our patient was first admitted with sudden peritonitis, which was considered to be possibly related to the acute superior mesenteric arterial dissection and intestinal ischaemia. During pre-operative CTA, we also found that the patient had severe coeliac axis stenosis. Considering that the patient did not have chronic abdominal pain or weight loss, we did not make the diagnosis of median arcuate ligament syndrome (MALS).

In order to improve his symptoms and increase the intestinal blood supply, we performed the first emergency operation, which was a minimally invasive endovascular repair to seal off the dissection, dilate the true lumen of the SMA and the stenosis of the coeliac axis. After that we performed laparoscopy, but no necrotic bowel was found. Abdominal pain was relieved postoperatively.

On his second admission, the patient presented with severe flatulence after meals, which was considered to be possibly related to intestinal ischaemia and hypomotility. Based on previous studies, inadequate splanchnic blood flow and ensuing local hypoxia result in microvascular injury, production of cytotoxic molecules, and cellular necrosis or apoptosis. Several intestinal cell types are vulnerable to damage during ischaemia– reperfusion injury, including epithelial cells and neurons and glial cells, which are part of the enteric nervous system (ENS). The ENS regulates intestinal motility, co-ordinates secretion, and contributes to gut immune function. Permanent damage to the ENS, through a variety of mechanisms, can result in longterm intestinal dysfunction.^10^,^11^

**Fig. 4:**
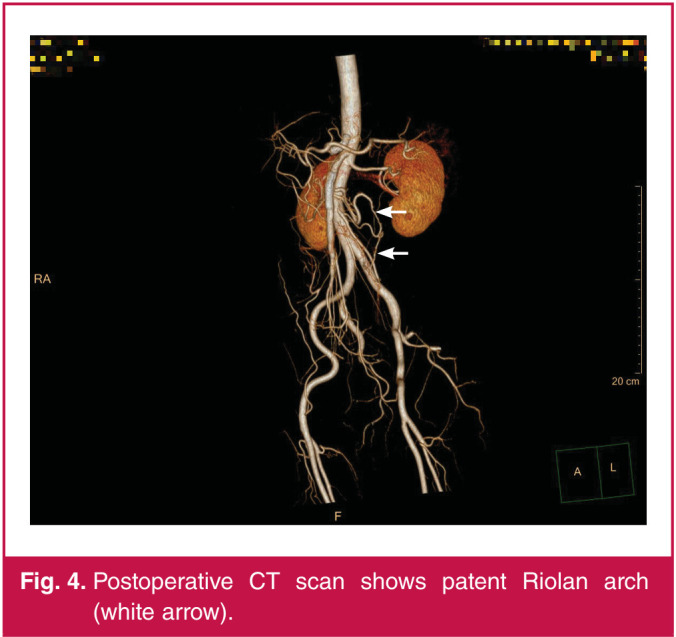
Postoperative CT scan shows patent Riolan arch (white arrow).

We performed a second-look laparoscopy and removed the abnormal intestine.^12^ The patient had a complete remission of symptoms postoperatively. After one year of follow up, the patient’s abdominal symptoms had not recurred and CTA showed good results (Fig. 4).

## Conclusion

Untreated superior mesenteric arterial dissection is related to high morbidity and mortality rates caused by progressive ischaemia of the bowel or aneurysm rupture.^13^ Among the variety of risk factors, condition of the branches is a very important factor affecting intestinal ischaemia. An invasive treatment approach was considered in patients with thrombosis of branches who developed increasingly severe pain after anticoagulant treatment.^14^ Endovascular stenting and laparoscopy are important minimally invasive treatments. 
